# The Good Wife

**Published:** 1873-01

**Authors:** 


### THE GOOD WIFE.

The Honorable John P. Kennedy, of Baltimore, who died recently in his seventy-seventh year, amid the regrets of many thousands who had read his numerous writings with delight, gave his wife the whole of his large property, and made her his sole executor, with this mention of her in his will:—

"I have reason to thank God for many blessings, for kind friends, worthy kinsmen, prosperous and contented life; for a cheerful temper, competence of worldly goods, a fair share of health, interrupted only by such alternations as have taught me the more to value it; for opportunities of public service, afforded me through the confidence of my fellow-townsmen, in more than one honorable trust; and, above all, for a home made dear to me by the affectionate and constant devotion of my wife, who has done every thing in her power to render me happy; whose rare virtues of mind and heart have given the most complete success to her endeavors."

May every reader of the Journal, who takes a wife during 1873, have reason to do by her at his death, and write of her in his will, as did John P. Kennedy.

